# Addressing Legal Risks in Public Health Communication Campaigns

**DOI:** 10.3390/ijerph23040481

**Published:** 2026-04-11

**Authors:** Kathleen Konopka, Aura Guerrero, Gianella Severini, Eric Crosbie

**Affiliations:** 1 Global Health Advocacy Incubator, Washington, DC 20005, USAaguerrero@advocacyincubator.org (A.G.); gseverini@tobaccofreekids.org (G.S.); 2 School of Public Health, University of Nevada Reno, 1664 Virginia St., Reno, NV 89557, USA; 3 Ozmen Institute for Global Studies, University of Nevada Reno, 1664 Virginia St., Reno, NV 89557, USA

**Keywords:** commercial determinants, legal risks, media campaigns, defamation, trademarks

## Abstract

**Highlights:**

**Public health relevance—How does this work relate to a public health issue?**

- Health advocates disseminate public health communication campaigns that inform populations about their health and rights and counter industry deception.
- Health-harming industries aggressively threaten and challenge public health communication campaigns to silence health advocates.

**Public health significance—Why is this work of significance to public health?**

- There is a general gap in the literature of commercial determinants of health that tends to focus on public health problems rather than solutions.
- Legal analysis is paramount to understanding how health campaigns can be designed and disseminated to the greatest effect while minimizing the risk of successful industry legal challenges.

**Public health implications—What are the key implications or messages for practitioners, policy makers and/or researchers in public health?**

- Health campaigns should encompass evidence-based facts, framed opinion or satire messages, and non-commercial intentions of informing people about their rights to health and access to information.
- Success in court can also set important precedents for advocates to disseminate important public health messages to the public.

**Abstract:**

The literature on the Commercial Determinants of Health has primarily focused on the political power and interventions of corporations versus countermeasures by civil society. However, there is a gap in the discourse concerning industry use of legal threats and actions to silence public health organizations and their public health communication campaigns (PHCCs). This legal review aims to understand legal challenges brought against PHCCs and provide best practices for effective campaigns while minimizing legal risk. We used jurisprudence in Mexico and the United States as proxies for civil and common law countries, supplemented by international law and principles to identify the best defenses and mitigation strategies against defamation and trademark infringement actions. Legal frameworks in these countries demonstrate the ability of aggressive evidence-based public health campaigns to withstand these legal challenges. Based on these legal protections, we recommend that advocates implement PHCCs that (1) contain facts that are true and substantiated with evidence, (2) frame messaging as opinion or satire, (3) highlight the non-commercial purpose of informing people about the impact of their consumption choices on health and well-being and (4) explain how the messaging advances the rights to health and access to information. A PHCC’s success in court can also set important precedents regarding the right of advocates to disseminate and the right of populations to receive important health messages. Following these recommendations and best practices, health advocates can minimize legal risk and continue to provide effective evidence-based PHCCs that promote and protect public health.

## 1. Background/Introduction

Noncommunicable diseases (NCDs) contribute to 74% of the global disease burden, including cancers, diabetes, chronic respiratory disease, neurocognitive disorders, and infertility [1]. Five commercial products, fossil fuels, tobacco, unhealthy food and beverages, chemicals, and alcohol, contribute to over 21 million deaths from NCDs globally each year—the largest contribution of any risk factors [1]. Corporations that manufacture, market, and sell these health-harming products are one of the primary disease vectors for the global spread of NCDs and mortality [2], with sales increasing particularly in low- and middle-income countries (LMICs) [3]. These products combined with the practices of their producers to maximize sales and profits by interfering with effective regulation and deceiving and exploiting consumers to alter public opinion are commonly referred to as the ‘Commercial Determinants of Health’ (CDoH) [4].

The World Health Organization (WHO) and other international bodies recommend a package of evidence-based public health policies, including taxes, advertising bans, and labeling on unhealthy products to reduce consumption of unhealthy products and promote public health [5]. Health advocates play a key role in supporting the adoption, implementation, and enforcement of these policies in part through the dissemination of public health communications campaigns (PHCCs) that inform populations about their health and rights and counter industry subterfuge and deception [6,7].

In response, health-harming industries have aggressively threatened and challenged governments, these PHCCs and the organizations that develop them [8,9,10]. Internal industry documents and public records have illustrated how companies have used legal threats and challenges to block, weaken and delay public health policies. These threats and challenges have been extended towards PHCCs designed to silence health advocates, their messages, and their support of public health policies. Overall, these legal threats and challenges have created a ‘chilling effect’ of preventing the diffusion of progressive and evidence-based PHCCs and public health policies [10,11].

While the CDoH literature has primarily focused on the relative impacts of corporate political and marketing activity versus public health advocacy on policymaking [12,13,14], there remains a general gap of focusing on public health problems rather than solutions. Additionally, there remains a specific gap concerning the industry tactic of threatening and initiating legal actions to silence PHCCs, and the legal protections health advocates possess to withstand these challenges and legally run their PHCCs. Legal analysis is paramount to understanding how PHCCs can be designed and disseminated to the greatest effect while minimizing the risk of successful industry legal challenges. This review addresses these gaps in the CDoH literature by examining two main legal challenges brought to silence these campaigns organized as follows: (1) the elements of and defenses to defamation, (2) the elements of and defenses to trademark infringement, (3) the human rights arguments that enhance protection for PHCCs, and (4) recommendations and tips to help health advocates develop and defend aggressive and effective PHCCs against legal threats and challenges.

## 2. Materials and Methods

The United States and Mexico were selected as common and civil law countries for this analysis because of the diversity of their legal systems and the highly developed bodies of jurisprudence in these areas. International law was intersected with the law of these countries given that many other countries, including those with less developed jurisprudence, often look to international guidance. The jurisprudence of each country was thoroughly researched using established online case law databases such as Westlaw and other country websites such as the Supreme Court of Mexico. The cases found were then compared to identify the similarities and differences between the proxy countries. Both bodies of law were then compared to international law to understand whether the jurisprudence was consistent with international guidance. The case study examples used were identified through information shared by GHAI’s global and local partners. They were then analyzed and synthesized to ensure their relevance to the topics addressed in the paper.

## 3. Results

### 3.1. The Ability of Aggressive Evidenced-Based PHCCs to Withstand Defamation Challenges

A primary legal threat and challenge that industry uses to silence and penalize health advocates for their PHCCs is defamation (Table 1). Defamation is broadly defined as either a written or spoken false statement of fact harming someone’s reputation. To prove defamation, a plaintiff generally must show that (1) the statement was false, (2) published, (3) made with fault (negligence), and (4) caused damage. When the statement is about a public figure, the plaintiff must meet a higher burden of fault by establishing “actual malice” (that the expresser knew the statement was false or acted with reckless disregard for truth) [15]. This section examines defamation under common, civil, and international law and available constitutional arguments and defenses to this claim.

Common law countries like the United States, United Kingdom, Caribbean countries, and many anglophone African countries rely heavily on judicial precedent to shape and interpret causes of action like defamation. Courts considering defamation actions are bound by prior court decisions within their jurisdiction and maintain the law as it has been shaped by this precedent under a doctrine known as stare decisis [15,16]. The First Amendment of the U.S. Constitution protects freedom of expression. This freedom may be abridged when demonstrably false statements of fact meet the other elements of defamation. However, statements of opinion and those that cannot “reasonably be interpreted as stating actual facts,” including those using satire, parody, or caricature to expose or criticize, are not actionable, because they are not factual [17,18]. This is true even if some readers mistakenly believe the statement conveys a fact, as long as the “hypothetical reasonable reader … (after time for reflection)” would understand the nature of the writing [17]. For example, in *Farah v. Esquire Magazine*, the U.S. Court of Appeals for the District of Colombia rejected a defamation claim against a published article that claimed that a book alleging President Barack Obama’s ineligibility to serve as president due to a lack of a U.S. birth certificate would be recalled and that buyers would receive a refund [17]. The *Farah* court found that “reasonable readers … would recognize the prominent indicia of satire in the Warren article,” based on a “number of humorous or outlandish details” included in the post [17]. These included an clearly fictitious book title and news story, sensationalistic and exclamatory headlines, and obviously false and contradictory admissions by the target of the criticism [17].

On the other hand, civil law countries like Mexico and many Latin American, continental European, and francophone African countries base their legal systems on comprehensive, codified statutes (e.g., Roman law), with judges primarily applying the written code as the source of law. The more detailed and comprehensive format of civil law statutes tends to provide less discretion for judicial interpretation, and judicial precedent may be less of a focal point than in common law countries. However, Mexico recognizes the legal action of defamation as including nearly identical elements and defenses to the cause of action recognized in the United States. Specifically, Mexican law notes that freedom of expression can be restricted if it harms a person’s dignity and honor. The Mexican Constitution protects the right of legal entities (“personas morales”), including corporations [19], from “infamy” (Article 22), an attack to a corporation’s good reputation or “good fame” [19].

While these protections exist, the harm resulting from alleged defamatory statements must be significant to override constitutional rights to expression. For example, in 2012, Act Health Promotion (ACT), a Brazilian non-profit organization, launched a mass media campaign that revelated how tobacco companies placed its tobacco products near chocolates other sweet products in stores that appeal to children [20]. Souza Cruz, a Brazilian subsidiary of British American Tobacco, filed a lawsuit with the Sixth Civil Chamber, Court of Appeal, State of Rio de Janeiro, to stop ACT’s PHCC, alleging that the campaign falsely accused the company of encouraging the illegal sale of cigarettes to minors [21]. The court rejected Souza Cruz’s defamation claim, finding that the company did not suffer harm sufficient to justify limiting freedom of expression. The court held, “The campaign has the main objective that the defendant’s products, namely cigarettes, are not sold alongside products intended for children and young people. This aligns with the company’s assertion that its products are not aimed at this demographic but rather at existing consumers. Upon review of the claim, there is no apparent threat to the defendant’s business activities” [21].

**Figure 1:**
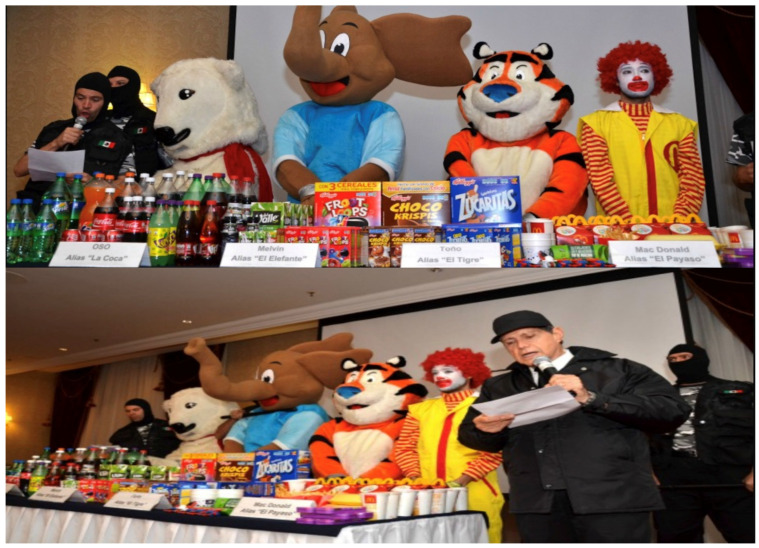
2013 El Poder del Consumidor campaign media activity addressing food industry predatory marketing to children that included a staged a mock criminal arrest of “the junk food cartel,” featuring mascots associated with products brands such as Zucaritas, Choco Krispis, and Coca-Cola. Reprinted with permission from El Poder del Consumidor. 2013, El Poder del Consumidor. Copyright.

As in the United States, true statements cannot be defamatory in Mexico. Additionally, the use of parody or satire to provide social commentary is an accepted vehicle that typically falls outside the reach of defamation. For example, in 2013, El Poder del Consumidor (EPC), a Mexican non-profit organization, ran a PHCC aimed at addressing food industry predatory marketing to children that included a staged a mock criminal arrest of “the junk food cartel,” featuring mascots associated with products and brands such as Zucaritas, Choco Krispies, and Coca-Cola (Figure 1). This PHCC avoided legal scrutiny by employing satire, including obviously fictitious arrests of characters or mascots, which no reasonable reader would believe was conveying a real event. EPC also ensured that all factual content in this campaign regarding products and health risks was accurate, evidence-based, and verifiable.

Both the United States and Mexico recognize that the right to freedom of expression more likely trumps the right to honor when a public figure or matter of public interest is concerned. The United States requires a higher standard of proof, “actual malice,” in order for statements regarding a public figure to be actionable, while a Mexican court has made clear, “issues of public interest must be uninhibited, robust and open, and may include vehement attacks, caustic and unpleasantly biting comments about public figures or, in general, ideas that may be received unfavorably by their recipients and public opinion (…)” [22].

International law seems to go a bit further on this point, specifically disfavoring defamation when it relates to a public figure or matter of public interest. The Inter-American Court of Human Rights found that a defamation action against a journalist for publishing statements regarding a public figure contravened article 13 of the Inter-American Convention, which protects freedom of thought and expression [23]. The Court stated that the obligation to prove the truth of the publication “produces a deterrent, frightening and inhibiting effect on all those who practice the profession which, in turn, impedes public debate on issues of interest to the society” [23].

Overall, truth is an absolute defense to defamation. Thus, it is paramount that facts in PHCCs be supported by evidence. Alternatively, campaigns could be clearly rooted in opinion or satire in a way that a reasonable reader would understand the statement to be non-factual. PHCCs are further protected under the law because they relate to public figures (corporations that have publicly promoted their brands and products) and a matter of public interest (health). Under national jurisprudence and international law, health advocates have the right to inform the public about the health risks associated with unhealthy products and marketing practices, without fear of reprisal through defamation claims.

### 3.2. Trademark Infringement

Another legal challenge that industry uses to silence and penalize health advocates for their PHCCs is trademark infringement. A trademark is a limited property right in a particular word, phrase or symbol (e.g., Coca-Cola wave). Trademarked logos and brands often are the single greatest representation of a company’s identity and are how a consumer will most easily recognize a particular product [24]. This section examines trademark infringement under common, civil, and international law and available constitutional arguments and defenses to this claim.

The U.S. has developed a robust body of jurisprudence that balances trademark rights of companies against the First Amendment right to non-commercial expression. The United States’ Lanham Act provides a cause of action for trademark infringement, but only when certain requirements are met [25]. Specifically, a petitioner must not only establish that it owns a valid and protectable mark used by the defendant but also bears the burden of showing that “the defendant’s use of the mark is likely to create confusion concerning the origin of goods or services” [26]. For example, in *Bosley Med. Inst. Inc. v. Kremer (2005)*, the U.S. Court of Appeals for the Ninth Circuit found the Lanham Act inapplicable and rejected all claims of infringement because Kremer was not using Bosley’s trademark to sell a competing product or service, but rather to express his opinions about Bosley’s products [27]. Limiting the Lanham Act to cases where a defendant is trying to profit from a plaintiff’s trademark is consistent with the Supreme Court’s view that “[a trademark’s] function is simply to designate the goods as the product of a particular trader and to protect his goodwill against the sale of another’s product as his” [27].

Court decisions can also strike the appropriate balance between trademark and First Amendment rights. For example, the *Bosley* court clarified the Lanham Act’s commercial-purpose requirement by ruling that “the First Amendment may offer little protection for a competitor who labels its commercial good with a confusingly similar mark, but trademark rights do not entitle the owner to quash an unauthorized use of the mark by another who is communicating ideas or expressing points of view” [27]. In brief, companies cannot use the Lanham Act “either as a shield from [a PHCC’s] criticism, or as a sword to shut [health advocates] up” [27].

This protection of non-commercial use of trademarks is further illustrated in *Mattel Inc. v. Walking Mountain Productions (2003)* [28]. In that case, the defendant created a photo series titled *Food Chain Barbie*, depicting Barbie dolls placed in various positions inside kitchen appliances to comment on the objectification of women and to critique the beauty standards embodied by the Barbie image. Mattel brought a federal trademark infringement lawsuit, but the Court rejected the claim. It found that the Barbie image and trademark were used not for commercial gain but for social commentary, constituting a parody protected by the First Amendment [28]. Although Barbie’s figure and face are highly creative works that generally receive strong protection, the defendant’s use of substantial portions of the image was justified given the parodic and critical purpose [28]. Finally, the Court held that the work posed no risk to Mattel’s commercial market, as the parody was unlikely to cause consumer confusion or impair the market for Barbie products [28]. This case is a clear example of the non-commercial use of trademarks, particularly when used to critique social conduct. It illustrates how trademarks can be employed strategically and effectively.

Similarly, in *U.S. Chamber of Commerce v. Jacques Servin et al. (2009)*, the political parody group known as the Yes Men held a mock press conference in which they impersonated representatives of the U.S. Chamber of Commerce and falsely announced that the Chamber was reversing its prior denial of climate change and would now support climate legislation [29]. Their purpose was to criticize the Chamber’s opposition to such legislation, raise public awareness about climate change, and pressure the Chamber to recognize it as a genuine issue. The Chamber responded by filing a lawsuit alleging trademark infringement based on the unauthorized use of its logo, but it voluntarily dismissed the suit before the motion to dismiss was decided [29]. The complaint underscored two key legal principles: that organizations seeking national prominence must tolerate a degree of public ridicule protected by the First Amendment and that, under the Lanham Act, restrictions on artistic works should apply only when the public’s interest in avoiding consumer confusion outweighs the public’s interest in free expression. Even where some degree of non-commercial consumer confusion may arise, the interest in protecting freedom of expression, particularly on matters of public interest, such as public health, should prevail.

Like the U.S., Mexico permits the non-commercial use of trademarks, especially when such use aims to inform consumers about a product’s ingredients, characteristics and associated risks. Read together, the Federal Law to Protect Industrial Property (*Ley Federal de Protección a la Propiedad Industrial*) [30] and the Federal Law for Consumer Protection (*Ley Federal de Protección al Consumidor*) [31] in Mexico make clear that trademark rights cannot be used to abridge consumers’ right to adequate and clear information about the different products available.

Consistent with the rationale for the non-commercial use exemption available in both the United States and Mexico, international legal instruments such as the Paris Convention of the World Intellectual Property Organization (WIPO) and the Agreement on Trade-Related Aspects of Intellectual Property Rights (TRIPS Agreement) of the World Trade Organization (WTO) make clear that trademark rights protect producers against unfair competition and to ensure consumers can reliably identify the product or service they want to obtain [32,33]. These concerns are not present in situations where public health advocates use logos to point out health concerns rather than to sell competing products.

**Figure 2:**
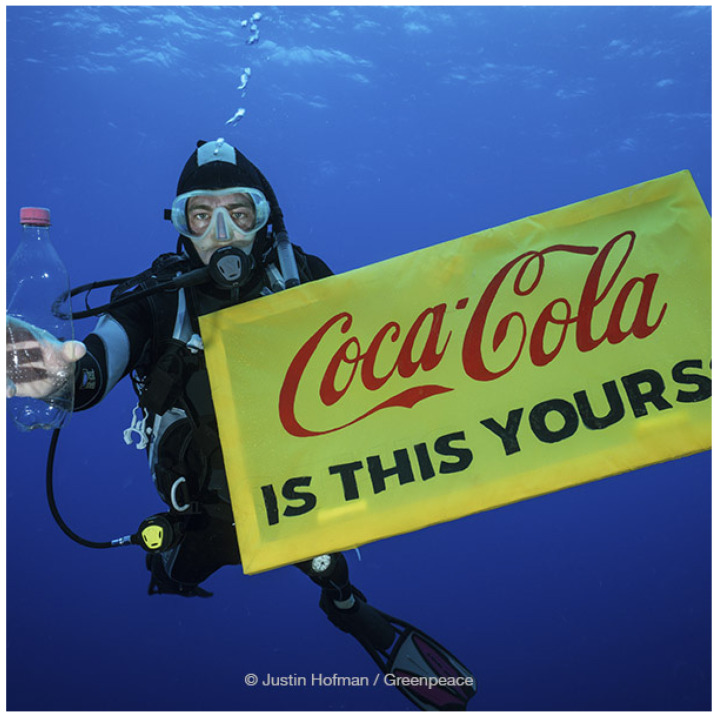
Greenpeace campaign addressing the climate crisis and Coca-Cola’s polluting of the environment globally that included a diver holding an empty Coca-Cola bottle and banner that reads “Coca-Cola: Is This Yours?”. Reprinted with permission from Greenpeace. 2018, Greenpeace.

Several public interest groups have been effectively used non-commercial trademarks to criticize industry harms to public health and the environment. For example, Greenpeace, a global environmental public interest organization, led a global campaign to address the climate crisis and Coca-Cola’s polluting of the environment globally, which included banners featuring Coca Cola’s iconic plastic bottle in various settings where plastic pollution is readily apparent to call for action on plastic and fossil fuel use and its impact on climate change (Figure 2) [34]. Many of these campaigns have been legally challenged by industries, but such actions have often been characterized by public opinion as intimidation lawsuits aimed at hindering public participation and silencing critics. These legal actions have been unsuccessful [35]. The non-commercial exemption to trademark infringement actions enables health advocates to use brands and logos in their PHCCs to identify the targets of their criticism, to call out company deception and misconduct, and to advocate for corporate responsibility and accountability for health and environmental harms.

### 3.3. Human Rights Protections for PHCCs

The right to health is enshrined in multiple international instruments (e.g., Articles 11 and 12 of the International Covenant on Economic, Social and Cultural Rights-ICESCR) [36] and national constitutions (e.g., Mexican constitution Article 4 and Colombian constitution Article 49). To fully realize this right, people require access to complete and accurate information. PHCCs further the right to health and information by counteracting industry deception, creating awareness of health risks, and enabling people to make informed decisions. Some jurisdictions have decided that PHCCs are entitled to additional protection because they further these fundamental human rights. This section examines these protections and the arguments that may be pursued under domestic and international law.

International human rights law codifies the right to the highest attainable standard of physical and mental health. The Committee on the Rights of the Child, which helps implement the Convention on the Rights of the Child, calls on States to ensure children’s right to health by combating and treating diseases, such as NCDs, and by implementing nutritional interventions, which should include the provision of information [37]. The Committee also stated the importance of non-State actors—including health advocates—in the provision of information on children’s health and its underlying determinants [37]. The Committee noted the responsibility of mass media organizations in promoting health and healthy lifestyles among children [37].

International instruments such as the Declaration on the Right and Responsibility of Individuals, Groups and Organs of Society specifically mention the right to information to ensure the health and wellbeing of individuals, such as women and children [38]. General Comment No. 14 of the Committee on Economic, Social, and Cultural Rights, which helps oversee the implementation of the ICESCR, states that the right to health depends on “the right to seek, receive and impart information and ideas on health-related matters” [39]. At a regional level, the Inter-American Court of Human Rights ruled that the right to access information is instrumental to other human rights, including health [40].

The WHO recommends the dissemination of “evidence-informed public campaigns and social marketing initiatives to inform and encourage consumers about healthy dietary practices” [41], including mass media campaigns that warn people of the dangers associated with consuming unhealthy products. For example, the WHO Framework Convention on Tobacco Control (FCTC) Partial Guidelines for Articles 9 and 10 state that Parties shall adopt and implement “effective measures for public disclosure of information about the toxic constituents of tobacco products” [42]. Furthermore, these guidelines note that the public should be informed about the health consequences, addictive nature and threat posed by the consumption of tobacco and recognize the important role health advocates play in raising awareness and disclosing this information [42].

Separate from its impact on the right to health, the right to seek, receive and impart information is protected under international and national law, including treaties like the International Covenant on Civil and Political Rights (ICCPR) and national constitutions [36]. The United Nations states that everyone has the right to “freely publish, impart or disseminate to others’ views, information and knowledge on all human rights and fundamental freedoms” [38]. The Inter-American Court has stated the importance of human rights defenders in promoting and protecting human rights through different activities, including monitoring, exposing and educating the population on human rights [40].

A particularly relevant example of these rights’ intersection with PHCCs arises from Colombia. In *Educar Consumidores v. the Superintendent of Industry and Commerce (SIC) (2017)*, the Constitutional Court ruled that administrative censorship of a campaign warning about the health impacts of sugary drinks violated both freedom of expression and the right of consumers to receive information, particularly where such information may have an impact on health [43]. Educar Consumidores, a public interest organization, initiated a mass media campaign entitled “Take Care of Your Life. Take It Seriously.” The campaign sought to “raise public awareness about the risk that high consumption of sugar-sweetened beverages poses to public health” [43]. Postobón, a major sugar-sweetened beverage company, filed an administrative complaint with the SIC, alleging that a mass media campaign was misleading because it did not explicitly state that only excessive consumption of products containing sugar causes health harm. The company further alleged that there were no scientific studies supporting the claim that sugar consumption has adverse health effects. SIC ordered the suspension of the campaign and required Educar Consumidores to submit any future advertising or public communication related to the consumption of sugar-sweetened beverages, across any media, for prior approval. The Constitutional Court reversed SIC’s order, finding it violated constitutional rights [43].

Similarly, in *Red PaPaz v. the Consortium of National Private Channels (CRC*, *2019)*, the Constitutional Court found that the refusal of private broadcasters to air a campaign against ultra-processed food marketing constituted unconstitutional censorship [44]. In both cases, the Constitutional Court underscored that non-commercial, evidence-based health communications are essential for democratic debate and deserve stronger constitutional protection than commercial advertising [43,44]. These decisions have enabled public interest organizations in Colombia to initiate and maintain aggressive PHCCs without further legal challenge.

## 4. Limitations

This study has a few limitations. While the consistency of our analysis across these common, civil and international law in these contexts supports a logical assumption of general applicability of our recommendations, we note that frameworks and results may vary in other jurisdictions. While the Americas were selected to provide a coherent and manageable comparative framework across common and civil law systems, we acknowledge that expanding the analysis globally would have introduced significant variability in legal frameworks, potentially affecting the depth and consistency of the assessment. We also recognize that legal protections, enforcement mechanisms, and institutional contexts may differ substantially in other regions, including Africa, Asia, and in more restrictive or authoritarian settings. These differences may influence both the interpretation and implementation of the legal principles discussed. Accordingly, while the analysis provides valuable insights, the applicability of the findings should be considered in light of regional legal and institutional variations, and further research would be needed to assess their transferability to other contexts.

## 5. Recommendations and Conclusions

Advocates enjoy considerable protection when disseminating truthful information on matters of public health and regarding companies that have put themselves in the public domain by marketing their products. This review demonstrates how PHCCs can navigate legal challenges and leverage rights-based arguments to protect and defend important information campaigns in the public interest.

We have several recommendations. First, all facts in a PHCC should be true and substantiated with evidence. Second, advocates could consider framing campaigns as opinion or satire because defamation is applicable only to statements reasonably interpreted by the reader as factual. Third, it is important to highlight the non-commercial purpose of PHCCs, which do not sell products but rather inform people about the impact of their consumption choices on health and well-being. Fourth, health advocates could explain how their messaging advances the rights to health and access to information. Finally, a PHCC’s success in court can set important precedents regarding the right of advocates to disseminate and the right of populations to receive important health messages. Following these recommendations and best practices, health advocates can minimize legal risk and continue to support aggressive evidence-based PHCCs that promote and protect public health.

## Figures and Tables

**Table 1:** Public health communication campaigns overcoming industry legal threats and challenges.

Country (Year)	Campaign (Organization)	Industry/Authority Response	Legal Issue Raised	Public Health Legal Defense	Outcome/Precedent
Brazil (2012)	ACT Health Promotion	Souza Cruz tobacco sued to stop campaign	Defamation	Freedom of expression, campaign did not name company, public interest	Court rejected injunction; upheld freedom of expression
Mexico (2013)	El Poder del Consumidor (EPC)	No legal action taken	Potential defamation or trademark use	Truthful information, satire, right to free expression	No lawsuit; campaign proceeded
Colombia (2017)	Educar Consumidores	Postóbon beverage company complaint and regulator (SIC) intervention	Alleged lack of truthfulness	Freedom of expression, right to information, right to health	Constitutional Court ruled in favor of public health groups
Colombia (2018–2019)	Red PaPaz	TV networks refused to air campaign	Censorship and chilling effect	Children’s rights, right to information, public health speech	Constitutional Court ordered dissemination
Global (multiple countries, various years)	Greenpeace	Major beverage corporations initiated legal challenges and threatened litigation against the campaigns	Trademark infringement, defamation, alleged misuse of brand and logos	Non-commercial use of trademarks; freedom of expression; public interest advocacy; right to criticize corporate conduct and expose environmental and health harms	Legal actions were unsuccessful; campaigns were widely recognized as legitimate; protection for non-commercial critical use of trademarks

## Data Availability

The original contributions presented in this study are included in the article. Further inquiries can be directed to the corresponding author.
